# Engineering microRNAs as multimodal cancer therapeutics

**DOI:** 10.1016/j.omtn.2026.103074

**Published:** 2026-09-05

**Authors:** Rui Kang, Daolin Tang

**Affiliations:** 1 Department of Surgery, UT Southwestern Medical Center, Dallas, TX, USA

## Main text

**Figure 1:**
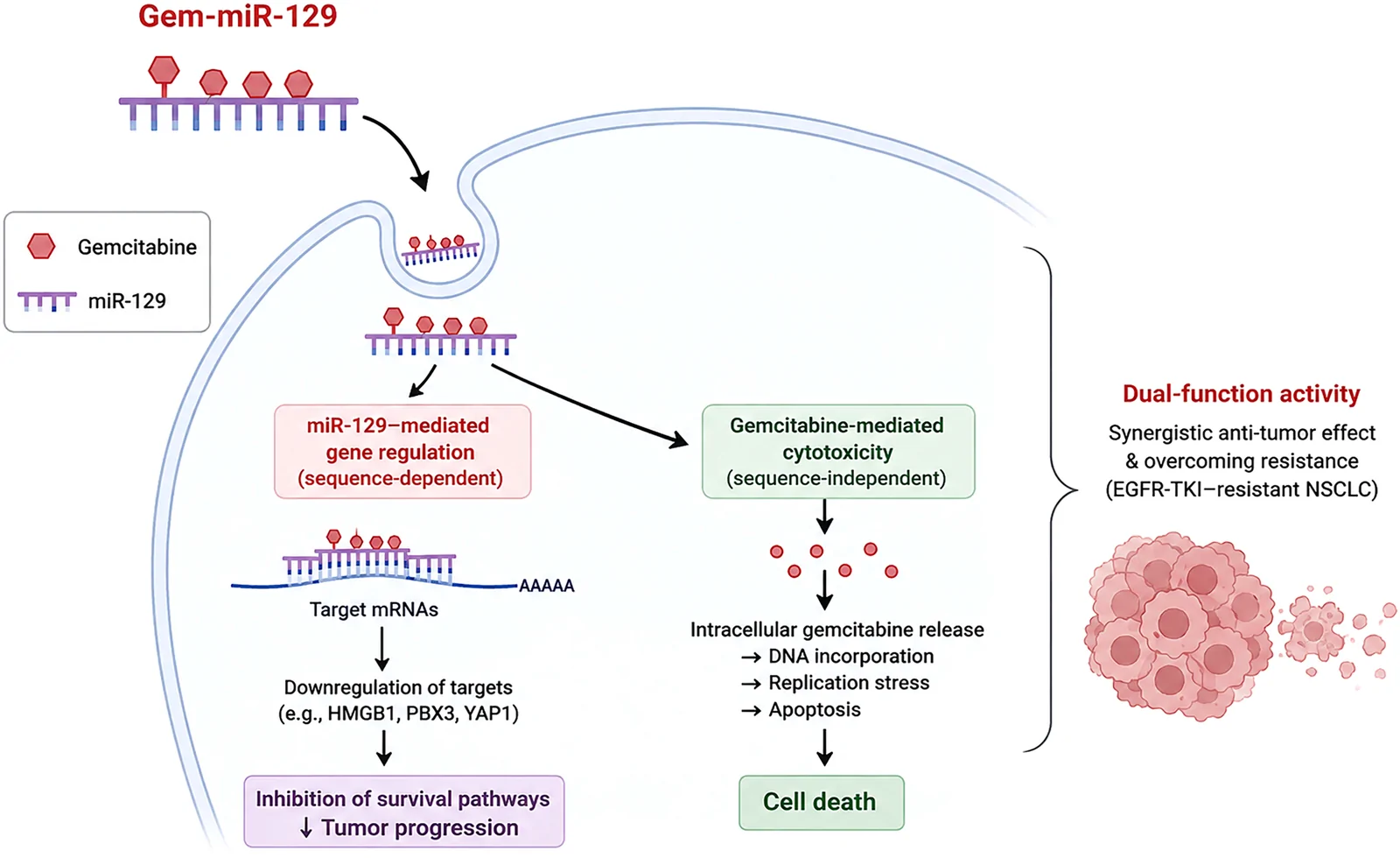
Proposed multimodal activity of Gem-miR-129 Gemcitabine is incorporated into the guide strand of the tumor-suppressive miRNA miR-129 to generate Gem-miR-129. The modified RNA retains miR-129-mediated regulation of oncogenic and resistance-associated proteins, including HMGB1, YAP1, and PBX3. Intracellular degradation of Gem-miR-129 may additionally release gemcitabine-containing metabolites that contribute to replication stress and S-phase accumulation, although this aspect of the mechanism has not yet been directly demonstrated. Together, these activities are associated with reduced proliferation and increased apoptosis in both parental and EGFR-TKI-resistant NSCLC cells.

Resistance to systemic therapy remains a major challenge in non-small cell lung cancer (NSCLC). Epidermal growth factor receptor (EGFR) tyrosine kinase inhibitors (TKIs) have improved outcomes for patients with EGFR-mutant tumors, but acquired resistance is common and can arise through diverse genetic and non-genetic mechanisms.^1^ This heterogeneity makes resistance difficult to address by targeting a single downstream pathway. In the previous issue of *Molecular Therapy Nucleic Acids*, Intriago and colleagues report a different approach by incorporating the nucleoside analog gemcitabine directly into the tumor-suppressive microRNA (miRNA) miR-129 (Figure 1).^2^ The resulting molecule, Gem-miR-129, retains activity against several miR-129-associated targets while showing low-nanomolar growth inhibition in genetically diverse NSCLC cells, including erlotinib- and osimertinib-resistant models. The study extends previous work on chemotherapy-modified miRNAs and further supports the concept that pharmacologically active nucleoside analogs can be incorporated into miRNA therapeutics while preserving their sequence-dependent regulatory function.^3^

miRNAs are attractive therapeutic molecules because a single miRNA can regulate multiple genes within related biological pathways.^4^ This property may be particularly useful in acquired drug resistance, where tumors frequently rely on overlapping mechanisms involving bypass signaling, altered cell states, enhanced survival, and impaired apoptosis. However, development of miRNA therapeutics has been limited by challenges including stability, cellular uptake, tissue distribution, and off-target effects.^5^ Chemical modifications can address some of these limitations, but they are generally intended to improve the properties of the RNA rather than provide an additional pharmacological effect.

Gem-miR-129 takes this idea in a different direction. The authors replaced cytidine residues in the miR-129 guide strand with gemcitabine while leaving the passenger strand unmodified.^2^ Previous studies from the same group established the feasibility of incorporating 5-fluorouracil into tumor-suppressive miRNAs, including miR-129.^6^^,^^7^ The present study extends this strategy to a nucleoside analog with a different mechanism of action, creating a molecule in which the RNA sequence and its chemical composition may both contribute to antitumor activity.

Gem-miR-129 showed potent activity across the NSCLC models examined. Low-nanomolar growth inhibition was observed in both *KRAS*-mutant and *EGFR*-mutant backgrounds, and activity was maintained in cell lines with acquired resistance to erlotinib or osimertinib.^2^ Gem-miR-129 also increased apoptosis and produced marked S-phase accumulation, consistent with replication stress. These findings suggest that its activity does not depend on continued sensitivity to EGFR inhibition and may therefore bypass at least some mechanisms responsible for TKI resistance.

The modified RNA also retained an important property of the parental miRNA. Gem-miR-129 reduced expression of high-mobility group box 1 (HMGB1), pre-B-cell leukemia homeobox 3 (PBX3), and Yes-associated protein 1 (YAP1), all of which have been associated with tumor progression or treatment resistance. HMGB1 is an established miR-129 target linked to autophagy-mediated resistance,^8^ whereas YAP1 has been implicated in EGFR-TKI tolerance and drug-persister states.^9^ The mechanism underlying YAP1 suppression remains to be fully defined, as both direct miR-129-mediated regulation and indirect effects related to cellular stress may contribute. Nevertheless, simultaneous effects on several resistance-associated pathways illustrate a potential advantage of retaining miRNA function within the modified molecule.

A relevant concern is whether incorporation of a nucleoside analog alters miRNA regulatory activity in unexpected ways. Exploratory RNA sequencing showed that the transcriptional response to Gem-miR-129 was largely predicted by the combined responses to unmodified miR-129 and gemcitabine, with relatively limited residual changes.^2^ Although these experiments cannot exclude individual off-target interactions, they provide initial evidence that gemcitabine substitution does not produce a broadly unrelated transcriptional response. Such analyses will be important for future chemically modified miRNA therapeutics, particularly when the modification itself has biological activity.

The *in vivo* experiments provide additional proof of concept. Gem-miR-129 reduced tumor burden and prolonged survival in a luciferase-expressing A549 (A549-Luc) xenograft model following intravenous or intraperitoneal administration.^2^ Gem-miR-129 also entered cultured NSCLC cells without a transfection reagent, a potentially useful property given the persistent delivery challenges of miRNA therapeutics. However, vehicle-free cellular uptake should be distinguished from vehicle-free systemic delivery. The animal experiments still used PEI-MAX transfection reagent to improve systemic exposure, stability, and delivery efficiency. Whether Gem-miR-129 can achieve sufficient stability, tumor accumulation, and tissue distribution without a carrier *in vivo* therefore remains unresolved.

Several mechanistic and translational questions remain. The proposed contribution of gemcitabine assumes that intracellular degradation of Gem-miR-129 generates gemcitabine-containing metabolites. Although the observed S-phase phenotype is consistent with gemcitabine pharmacology, formation of active metabolites has not been demonstrated directly. Measurement of 2′,2′-difluorodeoxycytidine triphosphate (dFdCTP), assessment of dependence on deoxycytidine kinase, and comparison with a stabilized miR-129 lacking gemcitabine would help define the contribution of conventional gemcitabine metabolism. The A549-Luc model is also not an acquired EGFR-TKI-resistant model, and the animal study did not include free gemcitabine, unmodified miR-129, or a non-targeting oligonucleotide formulated under the same conditions. Future studies in resistant models, together with pharmacokinetic, biodistribution, and toxicological analyses, will therefore be important. Because the current experiments used immunodeficient mice, potential effects on the tumor immune microenvironment also remain to be determined.

The broader significance of this work may lie less in Gem-miR-129 as an individual compound than in the design principle it illustrates. Chemical modifications of therapeutic RNAs are usually introduced to improve stability, affinity, or delivery; here, the incorporated nucleoside analog may itself contribute to pharmacological activity while the RNA retains multi-target regulatory function. Whether this strategy can be generalized to other tumor-suppressive miRNAs or nucleoside analogs remains to be determined. Although Gem-miR-129 is still at an early preclinical stage, the study provides evidence that nucleoside pharmacology and miRNA-mediated gene regulation can be combined within a single molecule, offering an approach worth further exploration for tumors driven by multiple coexisting resistance pathways.

## Acknowledgments

D.T. was supported by the National Institutes of Health (P50CA295495) and the American Cancer Society (MBGI-25-1423399-01-MBG). R.K. was supported by the National Institutes of Health (DK144077).

## Author contributions

D.T. and R.K. wrote and reviewed the manuscript.

## Declaration of interests

The authors declare no competing interests.

## Declaration of generative AI and AI-assisted technologies in the writing process

The figure was generated with the assistance of OpenAI image-generation tools and subsequently reviewed and refined by the authors.
